# Identifying host-specific amino acid signatures for influenza A viruses using an adjusted entropy measure

**DOI:** 10.1186/s12859-022-04885-7

**Published:** 2022-08-12

**Authors:** Yixiang Zhang, Kent M. Eskridge, Shunpu Zhang, Guoqing Lu

**Affiliations:** 1 Department of Statistics, University of Nebraska - Lincoln, Lincoln, NE USA; 2 Department of Statistics, University of Central Florida, Orlando, USA; 3 Department of Biology, University of Nebraska - Omaha, Omaha, NE USA

**Keywords:** Influenza A virus, Host specificity, Amino acid signatures, Adjusted entropy

## Abstract

**Background:**

Influenza A viruses (IAV) exhibit vast genetic mutability and have great zoonotic potential to infect avian and mammalian hosts and are known to be responsible for a number of pandemics. A key computational issue in influenza prevention and control is the identification of molecular signatures with cross-species transmission potential. We propose an adjusted entropy-based host-specific signature identification method that uses a similarity coefficient to incorporate the amino acid substitution information and improve the identification performance. Mutations in the polymerase genes (e.g., PB2) are known to play a major role in avian influenza virus adaptation to mammalian hosts. We thus focus on the analysis of PB2 protein sequences and identify host specific PB2 amino acid signatures.

**Results:**

Validation with a set of H5N1 PB2 sequences from 1996 to 2006 results in adjusted entropy having a 40% false negative discovery rate compared to a 60% false negative rate using unadjusted entropy. Simulations across different levels of sequence divergence show a false negative rate of no higher than 10% while unadjusted entropy ranged from 9 to 100%. In addition, under all levels of divergence adjusted entropy never had a false positive rate higher than 9%. Adjusted entropy also identifies important mutations in H1N1pdm PB2 previously identified in the literature that explain changes in divergence between 2008 and 2009 which unadjusted entropy could not identify.

**Conclusions:**

Based on these results, adjusted entropy provides a reliable and widely applicable host signature identification approach useful for IAV monitoring and vaccine development.

## Background

As members of the Orthomyxoviridae family, influenza A viruses (IAV) are negative-sense, single-stranded RNA viruses with a segmented genome that are occasionally deadly to humans and have been confirmed as causes of multiple pandemics resulting in large numbers of deaths [2]. Transmission to humans continually remains a global concern due to IAV’s vast genetic diversity, potential for rapid evolutionary change, ability to transmit among hosts and since they are widely circulating among migrating wild aquatic birds [3]. When a new IAV strain gains the ability to infect humans, it is usually not possible for the human immune system to respond fast enough to avoid severe infections, thus it is extremely important to monitor and predict IAV potential for transmission to humans. The establishment of IAV in humans is a multi-step process including transmission, replication, and adaptation which starts with sequence mutations. Since the amino acid sequences are usually one of the most accessible types of information from IAV databases, several computational methods have been developed to identify interspecies transmission candidate sites at the sequence level. The idea has been to use both phylogenetic and sequence alignment analyses to identify the essential amino acid mutations for proteins that are characteristic of the species origin of the sequences [4–6].

Several computational approaches have been considered for the host-specific signature identification. One approach is to measure the degree of uncertainty at a location using the proportion of amino acid residues of the aligned sequences from different hosts and decide whether it is a signature by comparing the dominant amino acid types. Chen et al. [7] first described such essential positions on aligned IAV sequences as host-specific genomic signatures and used an entropy measurement to locate avian-human signatures on each of 8 strains. Finkelstein et al. [8] introduced an approach to use statistical analyses of residue frequencies from pandemic H5N1 influenza viruses to identify persistent host markers. Another approach is to examine the strength of dependence/association between the amino acid mutations and hosts, using methods based on mutual information (MI) or the adjusted rand index (ARI) [9]. A similar idea was also used by Hu et al. [3] with the measurement based on the adjusted rand index (ARI) to evaluate the ability of characteristic locations to distinguish between different hosts. In addition, several machine learning approaches, such as neural nets, support vector machines, random forests and rule based modeling have been used for signature identification and predictions [4, 6, 10–13].

Despite the fact that these general approaches have proven to be useful in signature evaluation, all existing methods are based only on the proportion of amino acid residue types. With these approaches, all 20 standard amino acid types are implicitly assumed to be equally related to each other which is not a reasonable assumption [14–18]. Generally, the degree of uncertainty is directly based on how conserved the substitutions are within the given location since by definition, conservative substitutions vary little in terms of their biochemical properties.

Many methods have been proposed to understand the similarities among amino acid residues or to model their substitutions. The earliest approach was based on measurement or evaluation of various physical–chemical properties of amino acid residues [14]. Other methods that are based on empirical measurements of amino-acid replacement frequencies have been developed. Dayhoff et al.’s PAM model [19] was estimated using a counting approach and a similar model-based method has also been used by Jones et al. [16], Gonnet et al. [17] and Mueller et al. [20]. In 1992, Henikoff and Henikoff [18] introduced a direct way of counting amino-acid replacement frequencies, usually known as the BLOSUM scoring matrix, which avoids the extrapolation problems of the PAM model. More recently, many other amino acid replacement/substitution matrices have been described for sequence comparison and alignment and can also be considered in signature identifications [21–23].

We argue that approaches for host specific signature identification are improvable since they ignore the differences in similarities/substitution rates among the amino acid types. In this study, we propose a novel approach of adjusting the existing Shannon entropy measurement used for host-specific signature identification using both the proportions of amino acid residues and the similarities among them to identify the host-specific signatures. Specifically, we propose an adjustment coefficient derived from the BLOSUM matrix and incorporate the amino acid substitution information into the host-specific signature identification. This coefficient is used to construct an adjusted entropy measurement for signature identification. The adjustment is made using amino acids similarity/substitution rates, which we call the similarity coefficient (*SC*). The *SC* represents the average conservativeness of the substitutions among the amino acid residue types from a certain location. Our adjustment magnifies the entropy when amino acid substitutions have a lower level of similarity and reduces the entropy when a higher level of similarity is observed. We use simulated and real datasets to evaluate our method regarding host-specific signature identification as well as to compare the adjusted approach with Chen et al.’s [7] unadjusted entropy-based method. Mutations in the polymerase genes such as PB2 are known to play a major role in avian influenza virus adaptation to mammalian hosts. We thus focus on the analysis of PB2 protein sequences and identify host specific PB2 amino acid signatures. The results show that the proposed adjusted entropy method aids with monitoring essential IAV protein mutations which can provide useful information in virus monitoring and vaccine development.

## Results and discussion

### Method evaluation and threshold selection based on an H5N1 dataset

Table 1 shows an example of amino acid composition and corresponding proportions at two hypothetical positions of an alignment of 2000 IAV PB2 protein sequences, with Shannon entropy, *SC* (similarity coefficient) and adjusted entropy computed as described in the methods section.

**Table 1:** Amino acid composition, proposition, entropy, adjusted entropy and similarity coefficient (SC) at example positions of the PB2 protein sequence alignment

Attribute	Position 1 (n = 2000)	Position 2 (n = 2000)
Composition	1600^+^ Pro, 200 Phe and 200 Asn	1000 Tyr, 500 Phe and 500 Trp
Proportion (*P*_*i*_)	0.8^#^ Pro, 0.1 Phe and 0.1 Asn	0.5 Tyr, 0.25 Phe and 0.25 Trp
Entropy	0.639	1.040
*SC*^&^	0.625	3.605
Adjusted entropy*	1.022	0.288

^+^The number preceding the amino acid is the observed number of residues for that amino acid

^#^The number preceding the amino acid is the observed proportion of residues out of 2000 observed for that amino acid

^&^*SC* = Similarity Coefficient

*Adj. Entropy = Entropy/SC. See the methods section for a detailed explanation of adjusted entropy and a simple example

In this section, we compare the host-specific signature identification sensitivity performance using the unadjusted and adjusted entropy at two threshold values through the analysis of PB2 sequences. As the training data, we use all complete H5N1 PB2 sequences (avian, swine and human) from 1996 to 2006, which gives a dataset of 554 H5N1 PB2 sequences with the same length of 759 amino acids (AA) (strain names and accession numbers detailed in the data availability section). Similar to Chen and Shih [24], we exclude the 5 H5N1 avian influenza A sequences which were isolated from humans and use them as our validation dataset. Analysis of the 554 H5N1 PB2 sequences identified ten signatures using unadjusted entropy with a threshold of 0.33 where 9 were the same signatures found by Chen and Shih [24] and the remaining signature (674) was identified as a signature by Chen et al*.* [7] (Table 2). Using adjusted entropy and the same threshold (0.33), 22 signatures were found, of which 11 (BOLD) are new indicating improved sensitivity. Some of these new signatures could have been due to the threshold used by Chen and Shih [24]. Adjusting the threshold for adjusted entropy by using the SC of position 627 (SC = 2.2) gives 0.15 (0.33/2.2) as the new threshold. When applying this new threshold, only 7 positions were identified as signatures using adjusted entropy. Four of these positions were found by both unadjusted and adjusted entropy methods whereas three positions (BOLD) were not predicted by unadjusted entropy with the threshold of < 0.33 (Table 2).

**Table 2:** PB2 positions identified as host-specific signatures using unadjusted and adjusted entropy with two thresholds (0.33 and 0.15)

Method	Signatures
Unadjusted Entropy (< 0.33)	44 199 271 475 567 588 613 627 674* 702
Adjusted Entropy (< 0.33)	44 **67**^**+**^** 82 120 194** 199 **227** 271 **382 456 461 463** 475 **526** 567 588 613 627 674 **684 697** 702
Adjusted Entropy (< 0.15)	44 199 **227 382** 475 627 **697**

*Position 674 identified by Chen et al. [7]

^+^Bold figures indicates new signature identified by adjusted entropy

Another comparison of the two methods is based on the five different avian H5N1 influenza A viral strains isolated from humans. These are the same five strains excluded from our training dataset. Table 3 shows that the unadjusted method found one position (627) to be polymorphic, resulting in identifying two unique strains from the 5 for a false negative rate of 0.6 (3/5). However, Table 4 shows that the adjusted method identified 3 unique strains giving a 0.4 (2/5) false negative rate—a third smaller than the unadjusted method. In addition, Table 4 shows that for the adjusted method, out of the 7 identified signatures, we observed 2 mutations for 3 of the strains and 1 mutation for the remaining 2 strains whereas for unadjusted entropy, only 1 mutation was found out of the 10 identified signatures meaning that adjusted entropy was more efficient than unadjusted entropy.

**Table 3:** Signature positions and mutation patterns of PB2 identified by the unadjusted method

Strain	44	199	271	475	567	588	613	627	674	702	Mutations^+^
AAK49374(A/Hong Kong/482/97(H5N1))	A	**S**	T	L	E	A	V	E	A	K	1
AAK49375(A/Hong Kong/483/1997(H5N1))]	A	A	T	L	E	A	V	**K**	A	K	1
AAF74312(A/Hong Kong/483/1997(H5N1))]	A	A	T	L	E	A	V	**K**	A	K	1
ACZ45427(A/Hong Kong/483/1997(H5N1))]	A	A	T	L	E	A	V	**K**	A	K	1
CAB95862(A/Hong Kong/485/1997(H5N1))]	A	A	T	L	E	A	V	**K**	A	K	1

+Number of mutations

**Table 4:** Signature positions and mutation patterns of PB2 identified by the adjusted method

Strain	44	199	227	382	475	627	697	Mutations^+^
AAK49374(A/Hong Kong/482/97(H5N1))	A	**S**	V	I	L	E	L	1
AAK49375(A/Hong Kong/483/1997(H5N1))]	A	A	V	**V**	L	**K**	L	2
AAF74312(A/Hong Kong/483/1997(H5N1))]	A	A	V	**V**	L	**K**	L	2
ACZ45427(A/Hong Kong/483/1997(H5N1))]	A	A	V	**V**	L	**K**	L	2
CAB95862(A/Hong Kong/485/1997(H5N1))]	A	A	V	I	L	**K**	L	1

+Number of mutations

### Method evaluation based on simulation

To study the performance of the different methods, we develop a simulation algorithm to generate candidate sites based on small sets of real IAV sequences. The first part of the simulation process is to define “true positives” and “true negatives”. We start with real IAV sequence datasets of different divergent levels between different hosts. After alignment, we can directly define the “informative” starting point or “true positive” to be PB2 Pos-627 which is experimentally known as a host-specific signature (Chen et al*.* [7]). Avian influenza viruses most commonly possess a glutamine (E) at position 627 of PB2, while human viruses contain a lysine (K) at this position. An E627K substitution in PB2 confers the ability of an avian virus to replicate efficiently at low temperatures in vitro [7]. To identify the “true negatives”, we use the following process.

1. For each position in the alignment of training sequences, we find its dominant amino acid type and calculate its adjusted entropy within each host;
2. Select a position with different dominant amino acid types for different hosts;
3. For the selected positions from (2), consider those columns as “true negatives” if they are among those columns with the highest 20% of average adjusted entropy.

With these columns as the true positive and true negatives, we can simulate data to estimate the false positives and false negative rates using the following simulation process.

For false positive detection,

1. For each obtained “true negative” we can simulate data from a multinomial distribution using estimated amino acid proportions as the parameters. For example, for a column with 1600 Pro, 200 Phe and 200 Asn, we could obtain a multinomial distribution with parameters 0.8, 0.1 and 0.1 as the proportions of Pro, Phe and Asn, respectively.
2. Generate 1000 new columns with a length of 1000 for each “true negative” and its corresponding multinomial distribution;
3. Apply unadjusted and adjusted entropy methods to the generated columns and calculate the false positive rate.

Similarly, we can generate new columns from the multinomial distribution for the true positive column and calculate the false negative rate.

After the well-known “swine flu” pandemic in 2009, the IAV sequences derived from human and swine have considerably larger similarity values compared to those from before 2009. So, for the highly divergent training dataset, we choose to use all human/swine H1N1 PB2 sequences from only 2008. For the median divergent training dataset, we choose to use a subset of human/swine H1N1 PB2 sequences from 2000 to 2009 with the same sample size for each year. And for the less divergent training dataset, we choose to use all human/swine H1N1 PB2 sequences found in US from 2000 to 2009 with sequences from 2009 making nearly half of the dataset.

Table 5 shows that the adjusted entropy method has a much better performance in both sensitivity and specificity. Note that for the less divergent training dataset, no signature can be identified using the unadjusted entropy method which explains the 0% false positive rate and 100% false negative rate.

**Table 5:** False positive and false negative rates for both unadjusted and adjusted entropy methods

Training dataset	Unadjusted entropy		Adjusted entropy	
	False positive rate	False negative rate	False positive rate	False negative rate
Highly divergent	0.13	0.091	0.09	0
Median divergent	0	0.49	0	0
Less divergent	0	1	0	0.101

### Chronological analysis of signatures and related application based on an H1N1 dataset

The chronological analysis of genomic signatures was first conducted by Hu et al*.* [3] in 2014. The idea is to divide the IAV sequences isolated from different hosts (human, avian and swine) into different groups based on their collection years. According to Hu et al*.* [3], the number of avian-human host-specific signatures were relatively stable in the PB2 proteins across all time periods. But unlike the avian-human signatures, the numbers of swine-human signatures were markedly reduced during 1978–2009 and 2010–2013. One possible explanation provided by Hu et al*.* [3] is that the sequence-level genetic differences in the PB2 proteins between swine and human IAV might have decreased during those two time periods. But since the chronological groups of the IAV were only roughly divided (into 6 periods: 1902–1918, 1919–1957, 1958–1968, 1969–1977, 1978–2009 and 2010–2013), it is impossible to locate any exact change points. To better understand this phenomenon, we conduct a chronological analysis of swine-human host-specific signatures based on H1N1 PB2 data from 2004 to 2014 with each year as an observation. Table 6 shows the swine-human host-specific signatures identified for each year based on both adjusted and unadjusted entropy. According to our results, year 2009 resulted in the sudden drop of numbers of identified signatures. The average numbers of signatures in 2004 to 2008 is 19.8 based on unadjusted entropy method and 29.8 based on adjusted entropy method, which is close to the chronological signature numbers identified by Hu et al*.* [3] for the early three periods (1919–1957, 1958–1968 and 1969–1977 with numbers of signatures identified to be 20, 20 and 20, respectively). During the period from 2009 to 2014, the average numbers decreased to 0.17 using unadjusted entropy and 3.5 using adjusted entropy. We believe the sudden 2009 reduction resulted from the well-known H1N1 “swine flu” pandemic which cost an estimated 284,500 deaths [25]. We found from 2009 onwards, positions 54 and 315, then 66 and 731 are continually detected. The effects of these changes in viral proteins should be further investigated in vitro and in vivo.

**Table 6:** Swine-human signature positions identified using unadjusted (U) and adjusted (A) entropy for PB2 proteins during 2004–2014

Year	Type^+^	Signatures	n^++^
2004	U	9 44 81 91 105 114 199 354 355 395 399 411 447 475 490 491 547 567 627 702	20
2004	A	9 44 81 91 105 109 114 199 340 354 355 368 395 399 411 447 475 478 490 491 535 547 567 591 627 645 667 702	28
2005	U	44 64 81 91 105 114 199 354 395 399 411 447 475 490 491 567 627 702	18
2005	A	9 44 64 65 91 109 114 199 340 354 368 395 399 411 475 478 490 491 535 547 567 591 627 667 674 702	25
2006	U	9 44 81 91 114 199 354 355 395 399 411 447 475 490 491 547 567 627 702	19
2006	A	9 44 65 91 109 114 199 340 354 355 368 395 399 411 443 447 475 478 490 491 547 560 567 591 627 645 702	27
2007	U	9 44 64 105 106 109 114 199 354 355 368 395 399 447 475 490 491 547 567 627 661 674 702	23
2007	A	9 44 64 81 91 105 106 109 114 199 292 340 354 355 368 375 395 399 411 447 475 478 490 491 535 547 560 567 591 627 645 661 667 674 702	35
2008	U	9 44 64 81 105 114 354 355 395 399 447 475 490 491 547 567 627 674 702	19
2008	A	9 44 64 65 73 81 105 109 114 127 199 292 340 354 355 395 399 411 447 451 456 475 478 490 491 547 560 567 591 627 645 667 674 702	34
2009	U	NA	0
2009	A	54 315	2
2010	U	NA	0
2010	A	54	1
2011	U	NA	0
2011	A	54 315 354	3
2012	U	344	1
2012	A	54 315 344 354	4
2013	U	NA	0
2013	A	66 293 315 354 560 731	6
2014	U	NA	0
2014	A	66 315 354 560 731	5

^+^U = unadjusted, A = adjusted entropy

^++^n = number of signatures

To better understand the pandemic and the sudden drop in identified signatures, more mutation information is needed besides the number and position change in signatures identification. Table 7 shows the mutations from 2008 to 2010 at three positions: 354, 344 which are host-changing related markers suggested by Belanov et al*.* [26] and 560 which is with a host-specific signature change identified by adjusted entropy. According to the mutation investigation, no dominant amino acid change happened at position 344 during 2008–2010 and the mutation at 354 is with an amino acid change from “human-like” to “swine-like” type. The mutations I354L and V344M were likely acquired in May 2009 and the change could be related to the adaptation of the swine-origin H1N1 virus to the human host [26]. In contrast, position 560 identified as a host-specific signature by our adjusted entropy method is with an AA mutation from a “swine-like” to a “human-like”, which is more likely to be related to the H1N1 adaption to the human host. Note that position 560 is not detected by the unadjusted entropy method, which shows that the chronological analysis of signatures using adjusted entropy can be helpful for influenza surveillance and vaccine strain selection.

**Table 7:** PB2 amino acid mutations from 2008 to 2010 at three positions

Year	Position	Host	Dominant AA type	Identified as signature	
				Unadjusted entropy	Adjusted entropy
08	354	Swine	I	Yes	Yes
		Human	L		
	344	Swine	V	No	No
		Human	V		
	560	Swine	L	No	Yes
		Human	V		
09	354	Swine	I	No	No
		Human	I		
	344	Swine	V	No	No
		Human	V		
	560	Swine	V	No	No
		Human	V		
10	354	Swine	I	No	No
		Human	I		
	344	Swine	V	No	No
		Human	V		
	560	Swine	V	No	No
		Human	V		

## Conclusions

We demonstrate that adjusted entropy provides a reliable and widely applicable host signature identification approach useful for IAV monitoring. Validation with a set of H5N1 PB2 sequences from 1996 to 2006 results in adjusted entropy having a 40% false negative discovery rate compared to an 60% false negative rate using unadjusted entropy. Simulations across different levels of sequence divergence show a false negative rate of no higher than 10% while unadjusted entropy ranged from 9 to 100%. In addition, under all levels of divergence adjusted entropy never had a false positive rate higher than 9%. Adjusted entropy also identifies important mutations in H1N1pdm PB2 previously identified in the literature that explain changes in divergence between 2008 and 2009 which unadjusted entropy could not identify. The results show that adjusted entropy can aid with monitoring essential IAV protein mutations which can provide useful information in virus monitoring and vaccine development.

## Methods

### Adjusted entropy

The idea of signature identification is to evaluate each position for its potential to carry specific functions/properties. Entropy relates to uncertainty or disorder of a system and can be useful for signature identification since it is a measure of how conserved amino acid residues are at a location. Claude Shannon [27] defined information entropy as

$${\text{entropy}} = - \sum\limits_{all\;i} {(p_{i} \times \ln (p_{i} ))}$$

where *p*_*i*_ is the probability of observing the *i*th value of a random variable. Based on the composition of amino acids within each column of the IAV PB2 sequence alignment, entropy can be calculated to measure the uncertainty over the amino acid residues (*i* = 1–20) observed from each position of the aligned sequences with the same host. However, entropy for signature identification focuses only on the distribution of the proportion of amino acid residue types and ignores the similarities among the amino acid residues. We propose an adjusted entropy measurement incorporating both entropy and similarity such that adjusted entropy = Shannon entropy/similarity. The unadjusted entropy or Shannon entropy quantifies the uncertainty measurement and similarity, which is quantified by a similarity coefficient and measures the level of conservativeness for the given position. Our proposed host-specific signature identification method is similar to the entropy method introduced by Chen et al. [7] except we use adjusted entropy.

**Fig. 1:**
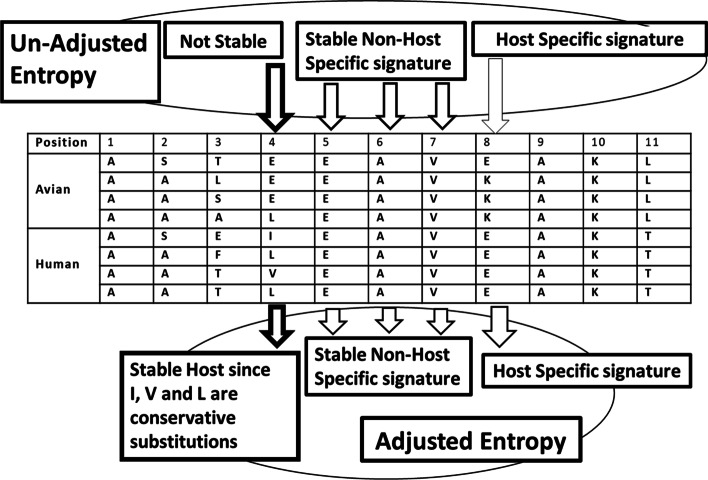
Host-specific signature identification method based on both adjusted and unadjusted (Shannon) entropy measurement

As a simple example, a portion of the alignment is shown in Fig. 1, which illustrates the difference between the proposed new method and the existing host-specific signature identification method based on the Shannon or unadjusted entropy. Using unadjusted entropy, mutation positions with lower entropy, *i.e.* with stable amino acid composition, are selected as potential signatures, while the positions with a higher entropy, *i.e.* with unstable/random amino acid composition, are ruled out, *e.g.* the nonstable positions (Fig. 1). Among the selected stable mutation positions 4 through 8, we can identify the host-specific signatures, based on a comparison of the dominant amino acid types from different hosts. Position 8 is identified as a host-specific signature by both methods. However, the adjusted entropy method identifies an additional host-specific signature, position 4, with a relatively high entropy but conservative. This example shows how identification of host-specific signatures can be improved by adjusting entropy using similarity. More details will be introduced in the next section about the calculation and application of the similarity coefficient (*SC*).

### Similarity coefficient (*SC*)

Adjusting entropy using a similarity coefficient magnifies the entropy of amino acids with non-conservative substitutions and reduces the entropy of those with conservative substitutions. With signature identification, the objective set of amino acids consists of *n* amino acid residues derived from a target position of the aligned IAV sequences where *n* denotes the number of sequences used for identification from a certain host. Assume these *n* amino acid residues have *m* different types (*m* = 1,2,…,20), where a “substitution” is defined as a pairwise replacement among the *m* amino acid types giving a total of *m*(*m*-1)/2 pairwise substitutions. For pairwise substitution, the conservative level may be quantified using a similarity score and the overall “similarity” among the *m* different types from the target position can be defined as the average of the *m*(*m* − 1)/2 possible pairs.

**Fig. 2:**
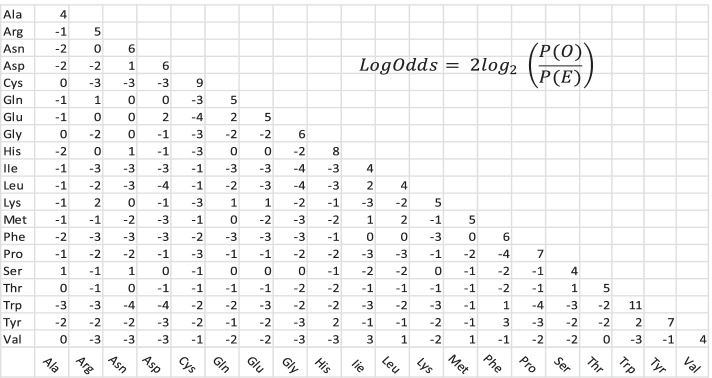
The BLOSUM62 scoring matrix for amino acid substitution. A table value for a particular pair of amino acids is the log odds defined as 2log_2_(*P(O)/P(E)*) where *P(O)* is the observed probability of occurrence of the pair and *P(E)* is the expected probability of occurrence of the pair assuming independence [18]. Similarities between amino acid pairs are based on log odds as described in the text

In this work, the log odds (Fig. 2) forms the basis of similarity coefficients (*SC*) among 20 standard amino acids where *P(O)* denotes the observed proportion of occurrences of the given residue pair and *P(E)* denotes the expected proportion of occurrences of the given pair due to chance alone [18]. Specifically, the BLOSUM 62 matrix (**BLO**cks **SU**bstitution **M**atrix; Fig. 2) is used as a score matrix with log odds values computed for all pairs of residues using frequencies from blocks of related proteins where two sequences within blocks were clustered as the same sequence if at least 62% of their aligned positions were identical [18]. BLOSUM scores have proven to be useful for the alignment of protein sequences since they provide information about conservativeness of substitutions among the 20 standard amino acids [28]. The BLOSUM matrix is a 20 × 20 scoring matrix providing negative scores (penalty) for non-conservative substitutions and positive scores (bonus) for conservative substitutions.

Based on the BLOSUM matrix, we obtain similarities between amino acid pairs by obtaining the difference between the observed and expected probabilities of each pair by applying the inverse function of the log-odds ratio. We define the similarity coefficient (*SC*) of a target position from the aligned IAV sequences as the inverse function (*f(x)* = *2*^*x/2*^) of the average centered BLOSUM62 scores. The formula is as follows:

$$\begin{aligned} SC & = 2^{{\left( {\frac{{\sum\limits_{k = 1, \ldots ,m} {\left( {\overline{{O(b_{k} )}} - \overline{{E(b_{k} )}} } \right)} }}{2m}} \right)}} \\ & = 2^{{\left( {\frac{1}{2m}\left( {\sum\limits_{k = 1, \ldots ,m} {\left( {\frac{{\sum\limits_{i = 1,...,m \, \& \, i \ne k} {b_{k,i} } }}{m - 1} - \frac{{\sum\limits_{j = 1,....,20 \, \& \, j \ne k} {b_{k,j} } }}{20 - 1}} \right)} } \right)} \right)}} \\ \end{aligned}$$

Here *m* denotes the number of amino acid residue types from the given position, *b*_*i,j*_ denotes the log odds score in cell *i,j* of the BLOSUM62 matrix, indicating the substitution probability of amino acid type *i* and *j* and $\overline{{O(b_{k} )}}$ and $\overline{{E(b_{k} )}}$ denote the observed and expected average BLOSUM62 score for amino acid type *k*, respectively. $\overline{{O(b_{k} )}}$ is calculated as the average log odds score of the observed AA (other than type *k*) paired with amino acid type *k* and $\overline{{E(b_{k} )}}$ is calculated as the average log odds score of all possible 19 AA (other than type *k*) paired with amino acid type *k*.

As an example of calculating the similarity coefficient *(SC)*, two amino acid compositions {Pro, Phe, Asn} and {Tyr, Trp, Phe} are assumed for the two given positions (*i.e.* two columns from the aligned amino acid sequences). *SC* is calculated for each position as follows:

$$\begin{array}{*{20}l} {b_{\Pr o,Phe} = - 4} \hfill & {b_{Tyr,Trp} = 2} \hfill \\ {b_{\Pr o,Asn} = - 2} \hfill & {b_{Tyr,Phe} = 3} \hfill \\ {b_{Phe,Asn} = - 3} \hfill & {b_{Phe,Trp} = 1} \hfill \\ {\overline{{E(b_{\Pr o} )}} = - 2.05} \hfill & {\overline{{E(b_{Tyr} )}} = - 1.21} \hfill \\ {\overline{{E(b_{Phe} )}} = - 1.63} \hfill & {\overline{{E(b_{Phe} )}} = - 1.63} \hfill \\ {\overline{{E(b_{Asn} )}} = - 1.26} \hfill & {\overline{{E(b_{Trp} )}} = - 2.26} \hfill \\ \end{array}$$

$$SC(Phe,Pro,Asn) = 2^{{\frac{{\left( {\frac{ - 4 - 2}{2} - ( - 2.05)} \right) + \left( {\frac{ - 4 - 3}{2} - ( - 1.63)} \right) + \left( {\frac{ - 3 - 2}{2} - ( - 1.26)} \right)}}{2*3}}} = 2^{ - 0.677} = 0.6255$$

$$SC(Phe,Tyr,Trp) = 2^{{\frac{{\left( {\frac{2 + 3}{2} - ( - 1.21)} \right) + \left( {\frac{3 + 1}{2} - ( - 1.63)} \right) + \left( {\frac{2 + 1}{2} - ( - 2.26)} \right)}}{2*3}}} = 2^{1.85} = 3.605$$

With this definition of *SC*, the positions with only 1 type of amino acid residue can be ignored since the Shannon entropy of such positions is 0 regardless of adjustment. Alternatively, when the positions contain all 20 standard amino acids, the *SC* will be 1 representing no adjustment to entropy, which is reasonable since the level of conservativeness is treated as “average”. In other cases, when more conservative substitutions are observed, *SC* will be greater than 1 representing a higher similarity level while if more non-conservative substitutions are observed, *SC* will be less than 1 representing a lower similarity level. Based on the BLOSUM62 matrix, most of the *SCs* are within a range from 0.1 to 10.

### Host-specific signature identification

The following is the process we use to identify signatures.

#### Identify the training dataset

Chen et al*.* [7] suggested using all sequences available as the training dataset for signature identification while in other studies (e.g. [3]), the training dataset can be selected based on different research goals. In this work, we illustrate our method with a simulated dataset and a partial real dataset and we also conduct analyses based on time and location.

#### Align sequences

In this step, all sequences from different hosts are aligned altogether. An option for us to balance the alignment based on different number of sequences of the 2 hosts is to use oversampling or undersampling which was introduced by Hu et al*.* [3]. We use MUSCLE [29] as the alignment algorithm which is used by both Chen et al*.* [7] and Hu et al*.* [3] in their signature identification methods.

#### Calculate entropy values for each position

Based on the composition of amino acid types within each column of the aligned IAV dataset, Shannon entropy is used to measure the uncertainty of the amino acid residues for each position of the aligned sequences within the same host, here either avian, swine or human. Then adjusted entropy is obtained by dividing entropy by the similarity coefficient:

$$adjusted\;entropy = - \sum\limits_{i = 1,...,20} {(p_{i} \times \ln (p_{i} ))} /SC$$

where each *p*_*i*_ denotes the proportion of the *i*th amino acid residue type. A larger similarity coefficient will reduce entropy and uncertainty.

#### Identify positions as potential signatures

Positions with entropy values below a threshold are identified as stable and considered potential signatures. Chen et al*.* [7] established a threshold by calculating the entropy of a certain position experimentlly known as a host-specific signature, specifically PB2-627, and used a threshold of 0.4 while Chen and Shih [24] suggested 0.33 based on a larger training dataset. We provide two different threshold values, one to maintain the same threshold and a second based on adjusted entropy of PB2-627.

#### For the selected positions, compare the dominant types of amino acid residues among different hosts to see if they can be identified as host-specific signatures

The positions with entropy values lower than the threshold with different dominant types of amino acid residues in different hosts are identified as signatures. These signatures may indicate that mutations at these locations related to a potential interspecies transmission.

As an illustration of how the *SC* and adjusted entropy may affect the signature identification, consider the two amino acid compositions {Pro, Phe, Asn} and {Tyr, Trp, Phe} considered in the above example. Further assume that each amino acid composition has 2000 total residues. Table 1 shows the composition and proportions for each position with the frequency or proportion of residues preceding each amino acid and Shannon entropy, *SC* and adjusted entropy computed as described above. Position 1 has a lower entropy value than position 2 but a much higher adjusted entropy since the 3 amino acid types for position 1 are quite “dissimilar” compared to the similarity among the 3 amino acid types of position 2. Based on adjusted entropy, the second position is considered as a preferred candidate host-specific signature while the unadjusted entropy method indicates the first position is preferred.

## Data Availability

The datasets generated and/or analyzed during the current study were downloaded from Influenza Virus Resource at the NCBI (https://www.ncbi.nlm.nih.gov/genomes/FLU/). See https://www.ncbi.nlm.nih.gov/pmc/articles/PMC4181488/bin/irv0008-0384-SD7.xlsx for the strain names and accession numbers of the viruses included in this study. The sequence information is available via the above link. It was published previously: World Health Organization/World Organisation for Animal Health/Food and Agriculture Organization (WHO/OIE/FAO) H5N1 Evolution Working Group. Revised and updated nomenclature for highly pathogenic avian influenza A (H5N1) viruses. Influenza Other Respir Viruses. 2014 May;8(3):384–8. https://doi.org/10.1111/irv.12230. Epub 2014 Jan 31. PMID: 24,483,237; PMCID: PMC4181488. Paper accession number: 66a45ce8-78e8-45fc-a912-c3f621f677d7.

## Abbreviations

AA: Amino acid
ARI: Adjusted rand index
BLOSUM: Blocks substitution matrix
IAV: Influenza A viruses
SC: Similarity coefficient
